# Development of an Orff music–based intervention program based on the PERMA model for children with cochlear implants: a Delphi study

**DOI:** 10.1186/s12887-026-07158-x

**Published:** 2026-06-15

**Authors:** Yujing Huang, Xiuqin Li, Wenxin Rao, Xinyue Sheng, Yuqing Zhang, Lulu Wang, Qinzhi Sun, Shasha, Biaoxin Zhang

**Affiliations:** 1 School of Nursing, Anhui Medical University, Hefei, Anhui China; 2 Department of Otorhinolaryngology-Head and Neck Surgery, The First Affiliated Hospital of Anhui Medical University, Hefei, Anhui China; 3 Jinwen Speech Rehabilitation Center, Hefei, Anhui China

**Keywords:** Cochlear implant, Children, Orff music, PERMA model, Delphi method

## Abstract

**Objective:**

To develop a PERMA-based Orff music intervention program for children with cochlear implants and to evaluate its preliminary effectiveness in rehabilitation.

**Methods:**

The intervention program was developed based on the PERMA model of positive psychology and Orff music therapy through a combination of literature review and the Delphi method. A panel of 15 experts in clinical medicine, rehabilitation, and nursing participated in two rounds of consultation to refine the program. Subsequently, a quasi-experimental study was conducted with 15 children who had cochlear implants to assess feasibility and potential effectiveness. Outcome measures included auditory, speech, and psychosocial indicators, evaluated before and after a 3-month intervention.

**Results:**

All experts completed both rounds of consultation, with importance ratings ranging from 4.2 to 5.0, indicating high consensus. The final intervention framework comprised 5 primary, 15 secondary, and 15 tertiary components. Following the 3-month intervention, significant improvements were observed across all outcome measures (*p* < 0.05). The program also demonstrated good acceptability and feasibility.

**Conclusions:**

The PERMA-based Orff music intervention showed preliminary effectiveness in improving auditory, speech, and psychosocial outcomes in children with cochlear implants. This multidimensional approach may provide a useful reference for developing comprehensive rehabilitation strategies in pediatric clinical practice. At the same time, the study results provide preliminary observational data on the feasibility of the intervention, which can serve as a reference for the design of future randomized controlled trials.

**Supplementary Information:**

The online version contains supplementary material available at 10.1186/s12887-026-07158-x.

## Introduction

Currently, nearly 400 million people worldwide are estimated to live with hearing impairment, including more than 30 million children with deafness. China has one of the largest populations of individuals with hearing loss globally [28]. According to reports from the World Health Organization, the incidence of childhood hearing impairment continues to rise annually, with approximately 0.5–5 per 1,000 newborns and infants affected by congenital or early-onset severe-to-profound sensorineural hearing loss [19].

Cochlear implantation (CI) has become the primary intervention for restoring auditory function in children with severe-to-profound hearing loss. Despite substantial advances in surgical techniques and device technology, postoperative outcomes remain highly variable, and long-term rehabilitation continues to present considerable challenges. Previous studies have shown that many children with cochlear implants experience delayed auditory and language development compared with their normal-hearing peers [17]. Cochlear implants bypass damaged cochlear hair cells and directly stimulate the auditory nerve through electrical signals; however, current devices process complex acoustic information using a limited number of electrode channels. Consequently, the spectral and temporal resolution of auditory input remains substantially reduced relative to normal auditory processing, which may impair sound localization, auditory discrimination, and auditory perception [25].

Beyond auditory and speech-related difficulties, children with cochlear implants may also experience psychosocial challenges. Communication difficulties in school settings and challenges related to social adaptation may contribute to negative psychological outcomes in some children with cochlear implants, including increased anxiety, heightened psychological stress, and reduced social confidence [17]. Increasing attention has therefore been directed toward socioemotional well-being, peer relationships, and social participation as important dimensions of CI rehabilitation [21]. These findings suggest that rehabilitation models focusing exclusively on auditory–verbal outcomes may be insufficient to support the broader developmental needs of children with cochlear implants.

Clinical guidelines for cochlear implantation emphasize that rehabilitation training is a crucial component of postoperative care, with the primary goal of promoting communication and language development, thereby helping children achieve developmental outcomes comparable to those of their peers with normal hearing [6]. Existing rehabilitation approaches include auditory–verbal therapy, visual reinforcement training, and music-based interventions. Among these, music therapy has received increasing attention because of its multisensory characteristics and strong motivational appeal. Previous studies have suggested that music training may improve speech perception and music perception abilities in children with cochlear implants [2]. In addition, the OPERA hypothesis proposes that intensive musical training combined with sustained attention and repeated auditory stimulation may enhance neural plasticity and facilitate language-related auditory processing [20].

Among music-based approaches, Orff music therapy may offer particular advantages for children with cochlear implants. Orff music emphasizes rhythmic movement, instrumental interaction, improvisation, and multisensory musical experiences [24]. By integrating auditory, visual, and motor activities, this approach encourages active participation and experiential learning and has been widely applied in both educational and therapeutic settings. Emerging evidence suggests that music-based interventions, including Orff-related approaches, may contribute to improvements in auditory perception, speech and language abilities, emotional engagement, and social interaction among children with cochlear implants [29]. Nevertheless, rehabilitation following cochlear implantation involves not only the recovery of auditory–speech abilities but also psychological adjustment and social adaptation. Interventions targeting isolated functional domains may therefore be inadequate to address the multidimensional developmental challenges faced by this population.

Positive psychology offers an additional theoretical perspective for comprehensive rehabilitation in children with cochlear implants. Rather than focusing solely on deficits or negative emotional experiences, positive psychology emphasizes the cultivation of individual strengths, positive emotions, and adaptive coping abilities [23]. In 2011, Martin E. P. Seligman introduced the PERMA model, which includes five core dimensions: positive emotion, engagement, relationships, meaning, and accomplishment [22]. Previous studies have shown that PERMA-based interventions can improve emotional well-being, resilience, and social functioning across a range of clinical populations [9].

Integrating music-based interventions with the PERMA framework may provide broader benefits for children with cochlear implants. Through activities such as music listening, rhythm-based movement, collaborative performance, and creative participation, children may develop greater attentional engagement, emotional involvement, motor coordination, and social connectedness, thereby supporting cognitive and psychosocial development [5]. However, most existing rehabilitation studies have primarily focused on quantitative auditory and speech-related outcomes, while relatively few have developed structured interventions grounded in explicit theoretical frameworks. In addition, current rehabilitation programs continue to prioritize auditory-verbal training, whereas psychosocial well-being and social adaptation receive comparatively less attention [10, 15]. To date, few studies have developed a comprehensive rehabilitation framework that systematically integrates Orff-based music therapy, auditory rehabilitation, and positive psychology principles.

Therefore, this study aimed to develop a multidimensional rehabilitation intervention program for children with cochlear implants by integrating Orff music therapy with the PERMA model through a systematic review of the relevant literature and expert consultation. Using the Delphi method, the study sought to construct a feasible and evidence-informed intervention framework to provide theoretical and practical guidance for future clinical rehabilitation and research.

## Materials and methods

### Establishment of the research team

The research team comprised one senior nursing supervisor (professor), three otolaryngology–head and neck surgery specialists, one rehabilitation therapist, and three Master of Nursing students. The nursing graduate students were responsible for literature retrieval, screening, and quality appraisal, while the supervisor oversaw the methodological rigor and scientific validity of the study. The otolaryngology specialists and rehabilitation therapists contributed to the extraction and synthesis of intervention-related content from the included literature. All team members were involved in the interpretation and discussion of the expert consultation findings.

### Literature search strategy

A systematic literature search was conducted in both Chinese and international databases, including China National Knowledge Infrastructure (CNKI), Wanfang Data, PubMed, Web of Science, and ScienceDirect. Relevant studies and clinical guidelines on Orff music therapy and postoperative auditory rehabilitation in children with cochlear implants were retrieved to identify current rehabilitation approaches, influencing factors, and music-based intervention strategies for pediatric cochlear implant users.

The Chinese search terms included “cochlear implantation,” “Orff music,” “music training,” “music therapy,” “children,” “rehabilitation training,” and “rehabilitation outcomes.” The English search strategy used combinations of the following terms: (“cochlear implantation” OR “cochlear implant”) AND (“Orff music” OR “music training” OR “music therapy”) AND “children” AND “rehabilitation.” Subject headings and free-text terms were combined to optimize the comprehensiveness of the search strategy.

### Literature inclusion and exclusion criteria

The inclusion criteria were established according to the PICOS framework.

Participants (P) included prelingually deaf children who met the indications specified in the 2013 Chinese Guidelines for Cochlear Implantation. Interventions (I) comprised Orff music training or other music-based interventions. Comparisons (C) involved conventional postoperative rehabilitation for cochlear implantation. Outcomes (O) included studies reporting clearly defined assessment tools related to postoperative auditory performance and subjective well-being. Study designs (S) were limited to randomized controlled trials and quasi-experimental studies.

The exclusion criteria were as follows: (1) reviews, conference abstracts, commentaries, or studies with incomplete data; (2) publications in languages other than Chinese or English; (3) duplicate publications; and (4) studies for which the full text was unavailable.

### Literature screening and quality assessment

Literature screening and quality appraisal were conducted independently by two researchers. Any discrepancies were resolved through discussion, and when consensus could not be reached, a third reviewer was consulted. The screening process consisted of three stages: removal of duplicate records, screening of titles and abstracts to exclude studies that did not meet the inclusion criteria, and full-text review of potentially eligible studies for final inclusion. The following information was extracted from each included study: first author, year of publication, music intervention approach, and primary outcome measures.

The methodological quality of randomized controlled trials was assessed using the modified Jadad scale [13]. Quasi-experimental studies were evaluated using the Joanna Briggs Institute (JBI) Critical Appraisal Checklist for Quasi-Experimental Studies, which includes nine appraisal items rated as “yes,” “no,” “unclear,” or “not applicable.” Final inclusion decisions were determined through group discussion among the research team.

### Development of the initial intervention protocol

Following quality appraisal, the research team systematically analyzed the intervention characteristics and reported outcomes of the included studies. Core components relevant to Orff music–based rehabilitation were extracted, compared, and synthesized. Drawing on the PERMA model of positive psychology and the theoretical principles of Orff music therapy, a preliminary draft of the intervention program was subsequently developed.

## Delphi method

### Expert selection

After the preliminary intervention protocol had been developed, 15 experts with relevant professional backgrounds were invited via email to participate in the Delphi consultation.

The expert inclusion criteria were as follows: (1) at least 10 years of clinical experience or a minimum of 5 years of research experience in nursing, otolaryngology–head and neck surgery, psychology, or related fields; (2) a bachelor’s degree or higher; (3) an intermediate or senior professional title; and (4) voluntary participation with informed consent.

### Questionnaire development

A Delphi consultation questionnaire was developed based on the preliminary music intervention program for children with cochlear implants. The questionnaire comprised three sections.

The first section provided instructions for completion, including the study background, research objectives, significance of the study, key concepts related to music therapy, and the timeline and submission requirements for the questionnaire.

The second section collected demographic and professional information from the experts, including age, gender, professional title, years of professional experience, area of specialization, degree of familiarity with the consultation content, and the basis for their judgments.

The third section consisted of the consultation items, covering the overall intervention framework, intervention themes, objectives, and specific program content. Each item was rated using a five-point Likert scale ranging from 1 (“not important”) to 5 (“very important”). In addition, open-ended comment sections were provided to allow experts to offer suggestions, revisions, or supplementary recommendations.

## Statistical methods

Statistical analyses were conducted using SPSS version 27.0. Continuous variables were presented as mean ± standard deviation (Mean ± SD), whereas categorical variables were summarized using frequencies and percentages. The normality of data distribution was assessed using the Shapiro–Wilk test before parametric analyses. All statistical tests were two-tailed, and a P value < 0.05 was considered statistically significant.

During the Delphi consultation process, the questionnaire response rate was used to assess expert engagement. Expert authority was evaluated using the authority coefficient (Cr), calculated from the judgment coefficient (Ca) and the familiarity coefficient (Cs). The familiarity coefficient was categorized as follows: 1.0 = highly familiar, 0.8 = relatively familiar, 0.6 = moderately familiar, 0.4 = somewhat unfamiliar, and 0.2 = unfamiliar.

The degree of consensus among experts was assessed using the coefficient of variation (CV) and Kendall’s coefficient of concordance (W). A CV value < 0.25 was considered indicative of acceptable agreement among expert opinions, while a Kendall’s W with *P* < 0.05 indicated statistically significant consistency among experts.

## Delphi expert findings

### Expert profile

The Delphi panel comprised 15 experts from multiple disciplines, including otolaryngology, rehabilitation medicine, clinical nursing, speech-language pathology, and music therapy. Most participants held senior or associate senior professional titles and had more than 10 years of clinical and/or research experience. The experts ranged in age from 33 to 59 years (mean 44.07 ± 6.68 years), and their years of professional experience ranged from 8 to 41 years (mean 22.12 ± 7.12 years). The demographic and professional characteristics of the experts are presented in Table 1.

**Table 1:** Characteristics of the Delphi expert panel (*N* = 15)

Characteristic	Participants, No. (%)
Sex	
Male	1 (6.7)
Female	14 (93.3)
Age, y	
30–39	4 (26.7)
40–49	8 (53.3)
50–60	3 (20.0)
Educational level	
Bachelor’s degree	8 (53.3)
Master’s degree	6 (40.0)
Doctoral degree	1 (6.7)
Professional field	
Otolaryngology	3 (20.0)
Rehabilitation medicine	2 (13.3)
Clinical nursing	4 (26.7)
Speech-language pathology	2 (13.3)
Music therapy	4 (26.7)
Professional title	
Intermediate	6 (40.0)
Associate senior	5 (33.3)
Senior	4 (26.7)
Clinical/academic experience, y	
10–15	3 (20.0)
16–20	3 (20.0)
> 20	9 (60.0)
Work setting	
Hospital	9 (60.0)
University	1 (6.7)
Rehabilitation institution	5 (33.3)

### Expert engagement and authority

A total of 15 questionnaires were distributed in each Delphi round, and all returned questionnaires were valid, resulting in a response rate of 100%. The experts demonstrated high authority, with familiarity (Cs) and judgment (Ca) coefficients of 0.95 and 0.83, respectively. The authority coefficients (Cr) were 0.87 in the first round and 0.89 in the second round.

### Coordination of expert opinions

In the first round of the Delphi consultation, the coefficient of variation (CV) ranged from 0.00 to 0.26, and the mean importance scores ranged from 4.20 ± 1.08 to 5.00 ± 0.00. In the second round, the CV values ranged from 0.00 to 0.21, with mean importance scores ranging from 4.13 ± 0.35 to 5.00 ± 0.00. Kendall’s coefficients of concordance (W) were 0.205 and 0.242 for the first and second rounds, respectively. The results indicated a statistically significant level of agreement among the experts (*P < 0.05*).

### Expert feedback and revision of the intervention program

Following the two rounds of Delphi consultation, the intervention protocol was further refined through iterative expert feedback and research team discussions. Revisions primarily involved clarifying activity descriptions, reorganizing the sequence of intervention sessions, simplifying tasks considered unsuitable for specific developmental stages, and strengthening the correspondence between Orff music activities and the PERMA dimensions.

More specifically, several activity instructions and terminologies were simplified to improve children’s comprehension and participation. Selected music activities and session arrangements were also adjusted to better facilitate rhythm perception, auditory attention, engagement, and rehabilitation-related objectives. In addition, tasks deemed excessively complex for certain age groups were modified to align with children’s auditory and cognitive developmental characteristics. This included reducing the complexity of singing and instrumental performance tasks and refining warm-up activities to better align with the intervention goals. Minor revisions were also made to improve the program’s structural organization and visual presentation.

After incorporation of the expert recommendations, the final Orff music intervention program for children with cochlear implants was established. The final framework comprised 5 primary domains, 15 secondary domains, and 15 tertiary intervention items. The core components of the intervention program are presented in Table 2, while the complete intervention content, item importance ratings, detailed expert comments, and item-level revisions are provided in Supplementary Material 5.

**Table 2:** Core components of the PERMA-based Orff music intervention program

Theme	Intervention Objective	Core Activities	PERMA Dimension
Theme 1. Sound Perception	To facilitate peer familiarity and establish positive emotional associations with sound	Environmental sound recognition; sound imitation; rhythm listening games	P (Positive Emotion): Positive emotional experiences are promoted through enjoyable exposure to environmental sounds and music.
Theme 2. Rhythm Sprites	To improve auditory–motor synchronization and rhythm discrimination through rhythmic movement activities	Body percussion training; movement-based rhythm exercises; group synchronization activities	E (Engagement): Rhythm-based activities enhance attentional focus and promote active participation.
Theme 3. Instrument Exploration	To promote auditory–tactile integration through instrument play and timbre perception	Group ensemble activities; turn-taking performance; peer cooperation games	R (Relationships): Cooperative music activities strengthen peer interaction and social connectedness.
Theme 4. Melody Rainbow	To enhance emotional expression, melodic perception, and auditory discrimination through singing and melodic activities	Singing activities; melodic imitation; emotional movement expression	A (Accomplishment): Progressive musical tasks and positive feedback enhance confidence and achievement experiences.
Theme 5. Story-Based Music Activities	To promote imagination, creativity, and language expression through music-based storytelling activities	Music storytelling; rhythmic role-play; drawing and scenario-based improvisation	M (Meaning): Integrated music and storytelling activities support meaning-making and expressive communication.

## Pre-experimental study of the music intervention program for children with cochlear implants

Following the two-round Delphi consultation, a preliminary pilot study was conducted to assess the feasibility, acceptability, and implementation process of the PERMA model–based Orff music intervention program. To minimize potential confounding influences, participants’ baseline characteristics and routine rehabilitation schedules were maintained as consistently as possible throughout the intervention period. The intervention was delivered by trained personnel according to a standardized protocol to ensure procedural consistency.

The primary purpose of this study was to develop and preliminarily evaluate the feasibility of the intervention program rather than to determine its clinical efficacy. Therefore, a single-group pre–post study design was adopted without inclusion of a conventional rehabilitation control group. During the study period, all participants continued to receive routine rehabilitation training, while the intervention duration, session frequency, and assessment procedures were standardized across participants. In addition, predefined inclusion and exclusion criteria were applied to reduce potential confounding factors related to age, cochlear implantation status, and cognitive functioning.

### Study participants

A convenience sampling method was used to recruit children receiving rehabilitation training at the Department of Otolaryngology–Head and Neck Surgery of a tertiary hospital in Anhui Province between December 1, 2025, and February 1, 2026. A total of 15 children with cochlear implants were enrolled in the study.

The inclusion criteria were as follows: (1) prelingually deaf children who met the indications specified in the 2013 Chinese Guidelines for Cochlear Implantation; and (2) children aged 6–12 years.

The exclusion criteria were: (1) children with medically diagnosed intellectual disabilities or other developmental disorders; and (2) children whose guardians declined participation or who were unable to complete the intervention for any reason.

### Intervention procedure

In addition to routine rehabilitation training and standard care, participants received a PERMA model–based Orff music intervention program. During the intervention, auditory activities were conducted in environments with minimal background noise. Therapists maintained face-to-face interaction to facilitate audiovisual integration, rhythmic stimuli were introduced progressively from simple to complex patterns, and auditory perception was supported through visual demonstrations and body movements.

Considering the attention span and tolerance of school-aged children with cochlear implants, each intervention session was designed to last approximately 20 min. To improve the reproducibility of the intervention protocol, two consecutive 20-min sessions (total duration approximately 40–45 min) are provided in Supplementary Material 4, including detailed descriptions of intervention procedures, activity arrangements, instructional strategies, and required materials.

Routine care included health education related to congenital hearing loss, prevention of common postoperative complications following cochlear implantation, and medication guidance. Outcome assessments were performed at baseline and three months after the intervention using the Auditory Assessment Scale, Speech Assessment Scale, Strengths and Difficulties Questionnaire, Children’s Social Anxiety Scale, and measures of subjective well-being.

### Outcome measures

Outcome measures

1. Meaningful Auditory Integration Scale (MAIS)

The Meaningful Auditory Integration Scale (MAIS) was used to assess children’s functional auditory behaviors in daily life. The scale consists of 10 items rated on a five-point scale, with higher scores indicating better auditory integration ability. The MAIS demonstrated good internal consistency, with a Cronbach’s α coefficient of 0.87.

(2) Meaningful Use of Speech Scale (MUSS)

The Meaningful Use of Speech Scale (MUSS) was used to evaluate speech use and short-term speech rehabilitation outcomes in children with cochlear implants. The scale comprises three domains, with total scores ranging from 0 to 40. Higher scores indicate better speech performance and rehabilitation outcomes.

(3) Subjective Well-Being

Subjective well-being was evaluated using measures of life satisfaction and emotional experience, including the Satisfaction with Life Scale and the Positive and Negative Affect Scale. Higher scores indicate greater life satisfaction or a higher frequency of the corresponding emotional experiences. The scales demonstrated acceptable reliability, with a Cronbach’s α coefficient of 0.71. Considering the young age of some participants and the potential communication and comprehension difficulties associated with hearing impairment, these questionnaires were completed by the children’s primary caregivers.

(4) Strengths and Difficulties Questionnaire (SDQ)

The Strengths and Difficulties Questionnaire (SDQ) was used to assess emotional and behavioral difficulties in children. The questionnaire contains 25 items rated on a three-point scale (0–2), with higher scores indicating more severe emotional and behavioral problems. The scale demonstrated good reliability, with a Cronbach’s α coefficient of 0.790.

(5) Children’s Social Anxiety Scale (SASC)

The Children’s Social Anxiety Scale (SASC) was used to assess social anxiety symptoms in children. The scale consists of 10 items covering dimensions such as fear of negative evaluation, social avoidance, and social distress. Higher scores indicate greater levels of social anxiety. The scale demonstrated good reliability, with a Cronbach’s α coefficient of 0.818.

### Intervention outcomes

All 15 children with cochlear implants completed the Orff music intervention program, resulting in a participation rate of 100%.

Detailed clinical information was collected for all participants, including age at cochlear implantation, duration of cochlear implant use before study enrollment, and implant and concurrent rehabilitation programs received during the study period. These variables were documented to improve the characterization of the study sample and to facilitate interpretation of the preliminary findings. Detailed results are presented in Table 3.

**Table 3:** Baseline characteristics of the participants (*N* = 15) data are presented as mean (SD) or n (%)

Characteristic	Number(%)
Age	
6–8 y	8 (53.3)
9–12 y	7 (46.7)
Sex	
Boys	9 (60.0)
Girls	6 (40.0)
Age at cochlear implantation, y	3.1 (1.8)
Cochlear implant experience, y	5.6 (2.7)
Implant side	
Unilateral	11 (73.3)
Bilateral	4 (26.7)
Primary rehabilitation modality	
Home-based rehabilitation	1 (6.7)
Institution-based rehabilitation	8 (53.3)
Combined home- and institution-based rehabilitation	6 (40.0)
Caregiver educational level	
High school or below	10 (66.7)
College degree	5 (33.3)
Monthly household income	
< 5000 RMB	3 (20.0)
5000–10 000 RMB	7 (46.7)
> 10 000 RMB	5 (33.3)

Following the 3-month intervention, improvements were observed across several auditory and psychosocial outcome measures compared with baseline, indicating preliminary positive changes. Detailed results are presented in Table 4. A 3-month follow-up period was selected because previous cochlear implant rehabilitation studies have demonstrated that early auditory, speech, and psychosocial changes may emerge during the initial postoperative rehabilitation stage [14]. Post-intervention scores for several outcome indicators showed improvement relative to baseline levels. However, given the small sample size and the absence of a control group, these findings should be interpreted as preliminary signals of potential benefit rather than definitive evidence of effectiveness. Further validation through larger controlled studies is warranted.

**Table 4:** Pre- and post-intervention difference test (3-Month intervention)

Outcome measure	Preintervention, Mean (SD)	Postintervention, Mean (SD)	t value	*P* value
Meaningful Auditory Usage Scale	24.60 (3.92)	27.20 (4.10)	7.01	< 0.001
Meaningful Speech Usage Scale	26.60 (3.24)	29.80 (3.46)	9.80	< 0.001
Subjective Well-being Total Score	32.10 (11.01)	38.90 (9.66)	−2.94	0.016
Strengths and Difficulties Total Score	14.00 (5.60)	10.90 (4.80)	5.13	0.001
Children’s Social Anxiety Total Score	6.70 (3.77)	5.00 (3.65)	4.02	0.003

## Discussion

Through a systematic literature review and two rounds of Delphi expert consultation, this study developed an Orff music intervention program for children with cochlear implants. The Delphi panel consisted of 15 experts from clinical medicine, nursing, and rehabilitation-related disciplines, all of whom had substantial clinical and academic experience. The questionnaire response rate reached 100% in both rounds, indicating a high level of expert engagement. The authority coefficients (Cr) were 0.87 and 0.89 across the two rounds, suggesting good credibility of expert judgments. The coefficients of variation ranged from 0.00 to 0.26 and 0.00–0.21 in the first and second rounds, respectively, indicating relatively stable expert opinions.

In addition, Kendall’s W values were 0.205 and 0.242 (*P < 0.05*). Although these coordination coefficients were relatively modest, they remained statistically significant and may reflect the multidisciplinary composition of the expert panel and the complexity of the intervention framework. Experts from different professional backgrounds, including medicine, nursing, and rehabilitation, contributed diverse perspectives regarding intervention content, implementation strategies, and expected outcomes. Therefore, the observed level of variation may not solely represent a limitation in consensus but may also reflect constructive interdisciplinary discussion and the integration of diverse professional viewpoints during the process of refining the intervention. The increase in Kendall’s W from the first to the second round further suggests that consensus improved as expert feedback was incorporated and the intervention framework was progressively refined.

Rehabilitation for children with cochlear implants extends beyond auditory and speech outcomes and includes psychosocial adaptation, peer interaction, emotional expression, and long-term adherence to therapy. The intervention developed in this study integrates the PERMA model of positive psychology with Orff music pedagogy. The PERMA framework emphasizes five dimensions—positive emotion, engagement, relationships, meaning, and accomplishment—and has been widely applied in health-related interventions to improve psychological adaptation and well-being [9, 27].

Emerging evidence suggests that Orff-based music training may facilitate auditory perception in children with hearing impairment [16]. Longitudinal neuroimaging evidence further suggests that structured musical training can induce neuroplastic changes in white matter pathways, reflecting enhanced interhemispheric connectivity between auditory and sensorimotor networks [12]. Such adaptations are thought to be driven by the demands of coordinated auditory processing, bimanual motor activity, and sensorimotor integration during music learning. Additional evidence indicates that music training strengthens auditory–motor integration networks and facilitates interhemispheric information transfer [11]. Moreover, even informal musical experiences such as singing have been associated with improvements in auditory perception and attentional processing in children with cochlear implants [26]. Beyond neurophysiological effects, Orff music activities may also contribute to psychosocial development. Group-based musical interaction, ensemble performance, synchronized movement, and musical storytelling provide structured opportunities for emotional expression, peer communication, engagement, and achievement experiences. These components align closely with the PERMA dimensions and may collectively support multidimensional rehabilitation outcomes.

From a developmental perspective, the intervention design is also consistent with the Special Educational Needs and Disability Code of Practice, which identifies communication and interaction, cognition and learning, social, emotional and mental health, and sensory/physical needs as key domains of difficulty in children [8]. Guided by this framework, the present study integrated Orff music activities with PERMA-based targets to address both functional and psychosocial rehabilitation needs.

Specifically, auditory and rhythm-based activities were used to facilitate positive emotional experiences and auditory–motor integration. Encouragement and structured reinforcement strategies were applied to enhance engagement and training motivation. Group music activities and cooperative performance tasks were designed to promote peer interaction and communication skills. In addition, musical narratives and thematic content were incorporated to broaden cognitive and emotional understanding, promote emotional engagement, and support self-expression during the intervention process [1]. Short-term, achievable goals combined with timely feedback created a positive reinforcement cycle, which may enhance rehabilitation adherence and sustained motivation.

The intervention adopted a progressive and structured curriculum, with task difficulty adjusted according to individual abilities to improve feasibility and participation. The pilot phase indicated good acceptability among participants; however, minor adjustments in pacing and task complexity were still required to accommodate individual variability. The intervention combined structured group sessions with home-based practice, encouraging parental involvement and promoting a family-centered rehabilitation model. Based on existing evidence, a frequency of twice-weekly sessions of 20–30 min was considered appropriate for this population. Activities included singing, rhythmic training, and body percussion to provide multisensory stimulation.

Previous studies have suggested that, in addition to conventional auditory–verbal therapy, music-based interventions may support receptive and expressive language development in children with cochlear implants [7]. Rhythmic and movement-based training has also been shown to align well with rehabilitation needs in this population. Consistent with prior literature, the present pilot findings suggest that auditory and speech development may be closely associated with emotional and behavioral functioning and social adaptation [3]. Improvements in communication ability may reduce social barriers, alleviate anxiety, and enhance emotional well-being and quality of life [4, 18].

Although some children showed positive trends in emotional and behavioral outcomes, prosocial behavior, and anxiety-related indicators, these findings should be interpreted with caution due to the small sample size and lack of a control group. Therefore, causal inferences regarding intervention efficacy cannot be established at this stage.

## Conclusion

In summary, this study developed a PERMA-based Orff music intervention program for children with cochlear implants through systematic literature review and Delphi expert consultation. The intervention emphasized progressive task design, positive reinforcement, and interactive music activities to enhance engagement and rehabilitation motivation. A family-involved model was also incorporated to support adherence and continuity of care.

The integration of PERMA principles with Orff music therapy may provide multidimensional support for auditory, speech, and psychosocial development in children with cochlear implants. However, due to methodological limitations—including small sample size, absence of a control group, and short follow-up duration—findings should be interpreted as preliminary evidence of feasibility rather than effectiveness.

Future research should include multicenter randomized controlled trials with larger samples and longer follow-up periods. More comprehensive outcome measures should also be applied to further evaluate the long-term clinical and neurodevelopmental impact of this intervention.

### Limitations

Several limitations should be acknowledged. First, the pilot study included only 15 children, limiting statistical power and generalizability. Second, convenience sampling from a single tertiary hospital may introduce selection bias. Third, the absence of a control group prevents attribution of observed changes solely to the intervention, as natural developmental progression and routine rehabilitation may also contribute.

Fourth, the follow-up period was limited to three months, which is insufficient to evaluate long-term developmental trajectories in cochlear implant rehabilitation. Fifth, outcome measures primarily focused on global functioning and did not include detailed domain-specific language assessments such as pragmatic language, suprasegmental features, or advanced auditory processing. Sixth, the literature search was limited to English and Chinese publications, introducing potential language bias. In addition, detailed information regarding cochlear implant brands and speech processor types was not systematically collected, which may have limited further exploration of device-related variability among participants. Finally, some psychosocial outcomes were based on caregiver-reported measures, which may introduce subjective bias related to parental perception and family environment. Therefore, psychosocial findings should be interpreted cautiously.

## Supplementary Information

Supplementary Material 1

Supplementary Material 2

Supplementary Material 3

Supplementary Material 4

Supplementary Material 5

## Data Availability

The datasets analyzed during the current study are not publicly available due to privacy and ethical restrictions, but are available from the corresponding author on reasonable request.
