# Supplementary material for: Development of an Orff music–based intervention program based on the PERMA model for children with cochlear implants: a Delphi study
Source: BMC Pediatr. 2026 Jun 15;26:713. doi: 10.1186/s12887-026-07158-x (PMC13445764; doi:10.1186/s12887-026-07158-x)
Supplement: Supplementary file 1 [file 12887_2026_7158_MOESM1_ESM.docx]

**Ethics Approval:**

This study was approved by the Ethics Committee of the First Affiliated Hospital of Anhui Medical University (Approval No. PJ-2025-10-59) and was registered with the China Clinical Trial Registry (Registration No. ChiCTR2500114602).

**Informed Consent Statement:**

Informed consent was obtained from all participants.

**Correspondence:**

Biaoxin Zhang, Email: zhangbiaoxin@ahmu.edu.cn
