# Supplementary material for: Development of an Orff music–based intervention program based on the PERMA model for children with cochlear implants: a Delphi study
Source: BMC Pediatr. 2026 Jun 15;26:713. doi: 10.1186/s12887-026-07158-x (PMC13445764; doi:10.1186/s12887-026-07158-x)
Supplement: Supplementary file 2 [file 12887_2026_7158_MOESM2_ESM.docx]

**Identification of studies via databases and registers**

Records identified from database searching (n= 2273):

Pub Med (n = 1841)

Web of Science (n = 35)

Science Direct (n= 378)

CNKI (n= 19)

Records removed before screening:

Duplicate records removed (n = 125)

**Identification**

Records excluded based on title or abstract (n = 1344):

- The intervention targets are not children with cochlear implants (n = 748)
- Not related to music-based intervention ( n = 596)

Records rescreening (n = 2148)

Reports not retrieved (n = 7)

Reports sought for retrieval

(n = 804)

**Screening**

Reports excluded ( n =785):

- Not in English or Chinese (n = 66);
- Lack of specific intervention measures (n = 522).
- Review articles, protocols, or conference abstracts (n = 132)
- Irrelevant outcomes or populations ( n = 65)

Reports assessed for eligibility

(n = 797)

Finally included in the literature

(n = 12)

**Included**

This figure shows the PRISMA flowchart, which details the study screening process. It includes the number of records identified, screened, assessed for eligibility, and included in the analysis.
