# Supplementary material for: Development of an Orff music–based intervention program based on the PERMA model for children with cochlear implants: a Delphi study
Source: BMC Pediatr. 2026 Jun 15;26:713. doi: 10.1186/s12887-026-07158-x (PMC13445764; doi:10.1186/s12887-026-07158-x)
Supplement: Supplementary file 4 [file 12887_2026_7158_MOESM4_ESM.docx]

**Supplementary Material 2**

**Sample PERMA-based Orff Music Session Plan**

**Theme**

**Song-Based Learning Activities Focused on Positive Self-Expression**

The intervention program follows a relatively standardized structure, though the musical themes and activity content vary from session to session. The representative lesson plan presented below is intended to demonstrate how this intervention framework is implemented in practice, thereby enhancing the transparency and replicability of the intervention.

**Session Duration**

20 min

The intervention sessions in the present study were conducted in approximately 20-minute formats. To improve intervention transparency and reproducibility, two representative sessions with different activity themes are presented below as examples of the intervention framework.

**Participants**

Children with cochlear implants aged 6–12 years

**Setting**

Group-based rehabilitation classroom

**Facilitators**

One music therapist and one rehabilitation nurse

**Table S1**

**Song Learning Session: Music-Guided Singing and Rhythmic Interaction**

| **Time** | **Intervention activity** | **Intended therapeutic response** | **Materials** | **PERMA domain** |
| --- | --- | --- | --- | --- |
| Pre-session preparation | Attendance was checked, and each child was greeted individually by the researcher to establish rapport and assess emotional readiness before the intervention session. | Facilitate emotional readiness and establish a positive therapeutic atmosphere. | Participant attendance list | Positive Emotion |
| 0-2 min | Warm-up： Children imitated a structured sequence of body percussion movements demonstrated in an instructional audiovisual clip and reinforced by the researcher, including two claps, one stomp, and alternating thigh patting synchronized with a steady musical beat. | Promote responsiveness to audiovisual rhythmic cues and facilitate gradual transition into active participation during the intervention session. | Audiovisual instructional clip demonstrating rhythmic body percussion sequences | Positive Emotion |
| 2-5 min | Play age-appropriate music that matches the children’s rehabilitation level (such as “Why Am I So Pretty?”) to introduce them to music and encourage their participation. | Promote auditory attention, emotional engagement, and responsiveness to musical stimuli. | Audio playback equipment and selected music tracks | Positive Emotion |
| 5-10 min | The researcher guided children to repeat short lyric phrases using a call-and-response singing format, followed by soft vocal imitation and synchronized group singing with background music. | Facilitate auditory–verbal engagement, vocal imitation, rhythmic speech production, and active participation through music-guided singing activities. | Audio playback equipment and selected music tracks | Engagement |
| 10-18 min | Children stood in a semicircle and performed synchronized rhythmic clapping activities guided by the researcher and accompanying music. The activity progressed from simple one-beat clapping patterns to alternating clap-and-step movement sequences synchronized with the musical rhythm. | Promote auditory–motor coordination, rhythmic synchronization, sustained attention, and active participation during group music activities. | Audio playback equipment and rhythmic music tracks | Engagement |
| 18-20 min | Review the lesson and assign a music activity for home. Encourage parents and children to use common household items—such as tapping bowls with chopsticks or shaking plastic bottles filled with beans—to create rhythmic sounds in time with the music. | Encourage parent–child interaction, reinforce auditory–verbal engagement, and extend music-based rhythmic participation into the home environment. | Household items | Relationships  Accomplishment |

**Abbreviation**: PERMA, Positive Emotion, Engagement, Relationships, Meaning, and Accomplishment.

**Table S2**

**Body Percussion and Creative Expression Session**

| **Time** | **Intervention activity** | **Intended therapeutic response** | **Materials** | **PERMA domain** |
| --- | --- | --- | --- | --- |
| Pre-session preparation | Attendance was checked, and each child was greeted individually by the researcher to establish rapport and assess emotional readiness before participation. | Facilitate emotional readiness and establish a positive therapeutic atmosphere. | Participant attendance list | Positive Emotion |
| 0-2 min | Children imitated a structured sequence of body percussion movements demonstrated in an instructional audiovisual clip and reinforced by the researcher, including two claps, three stomps, and alternating thigh patting synchronized with a steady musical beat. | Promote responsiveness to audiovisual rhythmic cues and facilitate gradual transition into active participation during the intervention session. | Audiovisual instructional clip demonstrating rhythmic body percussion sequences | Positive Emotion |
| 2-12 min | Under the researcher’s guidance, children participated in progressively structured body percussion activities synchronized with music. The rhythmic sequence included stomping, thigh patting, rhythmic clapping, and gesture-based movements (eg, thumbs-up gestures). The activity progressed from individual movement imitation to one-to-one guided interaction with the researcher, followed by small-group synchronized rhythmic activities requiring coordinated participation with peers. | Promote auditory–motor coordination, rhythmic synchronization, movement imitation, sustained attention, and cooperative social interaction during music-guided group activities. | Audio playback equipment and rhythmic music tracks | Engagement  Relationships |
| 12-18 min | The researchers played the song "How Come I'm So Good-Looking?" as background music while providing the children with A4 paper and colored pencils. Under the researchers' guidance, the children were asked to draw familiar themes related to self-image and positive emotions (such as "What do I look like in my mind?") and then briefly share their drawings with the whole class. | Encourage positive emotional expression, imaginative thinking, verbal interaction, and active engagement through music-guided creative activities. | Audio playback equipment, selected music tracks, A4 paper, and colored pencils | Positive Emotion Meaning |
| 18-20 min | Class review and assignment of a music activity for home. Parents are encouraged to practice the selected song, "Why Am I So Pretty," with their children at home. Specific activities include singing together and clapping in rhythm. | Encourage parent–child interaction, reinforce auditory–verbal engagement, and extend music-based rhythmic participation into the home environment. | Audio playback equipment and rhythmic music tracks | Relationships  Accomplishment |

**Abbreviation:** PERMA, Positive Emotion, Engagement, Relationships, Meaning, and Accomplishment.
