# Supplementary material for: Development of an Orff music–based intervention program based on the PERMA model for children with cochlear implants: a Delphi study
Source: BMC Pediatr. 2026 Jun 15;26:713. doi: 10.1186/s12887-026-07158-x (PMC13445764; doi:10.1186/s12887-026-07158-x)
Supplement: Supplementary file 5 [file 12887_2026_7158_MOESM5_ESM.docx]

**Appendix I**

**Summary of Experts' Comments**

Reviewer feedback (Round 1)：

Following expert feedback, the wording in Section 1.2.2 was modified from “text or images” to “physical objects or images” to enhance clarity and age-appropriate comprehension for children.

For Section 2.2.1, experts recommended allowing children to listen to and experience music independently during the initial listening phase, in order to better cultivate their perception and recognition of musical rhythm from an auditory perspective. It was also suggested that silent body movements be used instead of actions such as clapping and stomping to avoid interference with focused listening. In Section 2.1.4, “long–short rhythms” was revised to “fast–slow rhythms.”

For Section 3.1, experts recommended avoiding the simultaneous use of vocal singing and percussion instruments, as the combination of voice and instrumental sound may increase task difficulty for children. Furthermore, it was suggested that teachers should first present the song through singing alone, and then gradually increase task difficulty in subsequent stages. In addition, the original three-level framework (auditory, language, and psychological domains) was recommended to be simplified into a two-level structure.

Reviewer feedback (Round 2)：

Experts recommended adding professional terminology subtitles to the primary indicators to improve conceptual clarity. It was noted that the warm-up activity in Section 2.1.2 was not fully aligned with the intended objectives, and that the “Rhythm Sprites” module should primarily focus on rhythm perception and rhythm learning. Furthermore, it was suggested that graphical elements be added in Section 4.2.1 to enhance children’s engagement and comprehension.

**Full details of the intervention and the importance scores for each indicator**

Table. Expert Evaluation Scores and Variance Coefficients for Intervention Items

| Item | Importance Score | Coefficient of Variation |
| --- | --- | --- |
| 1. Theme 1: Sound Perception: Auditory Recognition and Environmental Sound Perception Based on Positive Emotion (PERMA-P). | 4.87±0.35 | 0.07 |
| 1.1 Cultivating Group Awareness and Positive Emotional Connections to Sounds | 4.33±0.49 | 0.11 |
| 1.1.1 Introducing the goals and formats of Orff music classes, establishing trust with students. | 4.67±0.49 | 0.10 |
| 1.1.2 Encouraging students to introduce themselves rhythmically through clapping, enhancing memory and interaction. | 4.60±0.63 | 0.14 |
| 1.2 Identifying Environmental Sounds and Simple Melodies. | 4.73±0.46 | 0.10 |
| 1.2.1 Students listen and mimic sounds through body movements, such as the "Rain" activity, which involves recognizing sound intensity and corresponding actions. | 5.00±0.00 | 0.00 |
| 1.2.2 Matching sounds with corresponding images, involving a family extension task to explore sounds at home. | 5.00±0.00 | 0.00 |
| 1.3 Auditory: Use of instruments (shakers, rain tubes) to simulate environmental sounds and enhance sound perception. | 4.53±0.83 | 0.18 |
| 2. Theme 2: Rhythm Spirit: Rhythm Training and Body Movement Based on Engagement (PERMA-E). | 4.67±0.49 | 0.10 |
| 2.1 Enhancing Synchronization of Auditory and Motor Skills through Rhythm. | 5.00±0.00 | 0.00 |
| 2.1.1 Students perceive rhythm changes through body movements, adjusting their speed to match rhythm variations. | 4.67±0.49 | 0.10 |
| 2.1.2 Combining rhythm exercises with life scenarios, such as the "Qingming Festival" activity with rain sounds. | 4.73±0.46 | 0.10 |
| 2.2 Enhancing Engagement and Self-Identity through Learning Songs. | 4.73±0.46 | 0.10 |
| 2.2.1 Gradually increasing the difficulty of learning songs, progressing from listening to singing and adding movements. | 4.73±0.46 | 0.10 |
| 2.2.2 Organizing group performances and rewarding students to build confidence and a sense of achievement. | 4.87±0.35 | 0.07 |
| 2.3 Auditory: Improving Consistency in Auditory and Motor Skills and Rhythm Recognition. | 4.53±0.64 | 0.14 |
| 3. Theme 3: Instrument Magic: Instrument Learning and Sound Imitation Based on Relationships (PERMA-R). | 4.73±0.46 | 0.10 |
| 3.1 Using Instrumental Play and Sound Imitation to Enhance Auditory-Tactile Integration and Promote Social Interaction. | 4.87±0.35 | 0.07 |
| 3.1.1 Pre-class warm-up games to help students familiarize themselves with instruments, promoting tactile and auditory connections. | 4.67±0.62 | 0.13 |
| 3.2 Increasing Physical Activity and Strengthening Auditory Memory. | 4.60±0.83 | 0.18 |
| 3.2.1 Introducing rhythm exercises through a thematic activity (e.g., "Harvest") and gradually increasing difficulty. | 4.33±0.62 | 0.14 |
| 3.3 Auditory: Matching and Identifying Simple Instrument Sounds. | 4.60±0.63 | 0.14 |
| 4. Theme 4: Melody Rainbow: Melody Listening and Song Learning Based on Achievement (PERMA-A). | 4.73±0.46 | 0.10 |
| 4.1 Enhancing Emotional Expression and Perception through Melody and Song Learning. | 4.73±0.60 | 0.13 |
| 4.1.1 Students recognize and express emotions through facial expressions and body movements in response to different melodies. | 4.13±0.35 | 0.09 |
| 4.2 Enhancing Achievement and Joy through Group Song Learning. | 4.67±0.49 | 0.10 |
| 4.2.1 Using children's songs and visual aids, students progress through stages of singing and understanding song content. | 4.73±0.46 | 0.10 |
| 4.2.2 Organizing group performances to encourage participation, rewarding students, and boosting their self-esteem and sense of accomplishment. | 4.13±0.35 | 0.09 |
| 4.3 Auditory: Improving Pitch and Tone Recognition. | 4.20±0.41 | 0.10 |
| 5. Theme 5: Story Context: Music Exploration through Improvisation Based on Meaning (PERMA-M). | 4.80±0.41 | 0.09 |
| 5.1 Enhancing Imagination and Creativity through Drawing, Rhythm Imitation, and Scenario Adaptation. | 4.80±0.56 | 0.12 |
| 5.1.1 Pre-class warm-up games to encourage creative expression through drawing. | 4.20±0.41 | 0.10 |
| 5.2 Converting Abstract Words into Musical Symbols. | 4.33±0.82 | 0.19 |
| 5.2.1 Students listen to rhythmic sequences and add key words to complete phrases or create new ones. | 4.40±0.91 | 0.21 |
| 5.3 Auditory: Strengthening Perception of Sound and Language Structure. | 4.20±0.41 | 0.10 |
